# A hybrid evolution strategies algorithm for non-permutation flow shop scheduling problems

**DOI:** 10.1038/s41598-025-88124-y

**Published:** 2025-04-07

**Authors:** Bilal Khurshid, Shahid Maqsood, Muhammad Salman Habib, Muhammad Omair, Seung-June Hwang

**Affiliations:** 1 Department of Industrial Engineering, University of Engineering and Technology, Peshawar, 25000 Pakistan; 2 Department of Industrial Engineering, Jalozai Campus, University of Engineering and Technology, Peshawar, 25000 Pakistan; 3 Institute of Knowledge Services, Center for Creative Convergence Education, Hanyang University ERICA Campus, Ansan-si, Gyeonggi-do 15588 South Korea; 4 Department of Materials and Production, Aalborg University, Aalborg Øst, 9220 Denmark; 5 College of Business and Economics, Hanyang University ERICA Campus, Ansan-si, Gyeonggi-do 15588 South Korea

**Keywords:** Non-permutation flow shop scheduling problems, Hybrid evolution strategies, Local search technique, Makespan, Mechanical engineering, Software

## Abstract

Flow shop scheduling has garnered significant attention from researchers over the past ten years, establishing itself as a prominent area of study within the field of scheduling. Nevertheless, there exists a paucity of research dedicated to addressing Non-Permutation Flow Shop Scheduling Problems. In this study, a Hybrid Evolution Strategies (HES) is suggested by combining the exploitation ability of Nawaz, Enscore, and Ham (NEH) Heuristic, the exploration ability of Improved Evolution Strategies (IES), and a Local Search Technique to minimize the makespan of NPFSSP. The primary solution is produced through the NEH Heuristic, serving as a foundational solution for the IES. The IES is applied in two stages, in the first stage it improves the permutation sequence found from the NEH heuristic. In the second stage of the IES, the permutation sequence on the first 40% of machines is fixed as found in the first stage. The sequence on the last 60% of machines is altered only so that the makespan is minimized and a good non-permutation sequence is found. Recombination and mutation are the main genetic operators in IES. For recombination in IES, 16 offspring are generated randomly from a single parent. The Quad swap mutation operator is employed in the IES to optimize the utilization of the solution space while minimizing computational time. To prevent trapping in local minima, a Local Search Technique is integrated into the IES algorithm, which guides solutions to less explored areas. Computational analyses indicate that HES exhibits superior performance regarding solution quality, computational efficiency, and robustness.

## Introduction

In today’s competitive business environment, precise scheduling is the best tool for survival, success, and growth. Scheduling is the optimal allocation of resources. In a manufacturing environment, when all machines have the same processing sequence and a job has to be processed once on each machine it is termed as flow shop. Although flow shop has many applications i.e. manufacturing industry, process industry, automobile industry, pharmaceutical industry, glass industry, and steel industry, few practical techniques are proposed for its practical implementation. Flow Shop Scheduling Problems (FSSP) with more than two machines are NP-hard problems^1^. In the context of Flow Shop Scheduling Problems (FSSP), a job consists of “m” operations, each executed on a distinct machine. The sequence of jobs on each machine is optimized to achieve the desired objective function. The major types of FSSP are Permutation Flow Shop Scheduling Problems (PFSSP), Non-Permutation Flow Shop Scheduling Problems (NPFSSP), No-Wait Flow Shop Scheduling Problems (NWFSSP), Blocking Flow Shop Scheduling Problems (BFSSP), Hybrid Flow Shop Scheduling Problems, among others.

In the context of PFSSP, every machine follows an identical processing sequence, allowing any job to be executed on any of the available machines. Conversely, in NPFSSP, while all machines maintain the same processing sequence, the order of jobs assigned to each machine varies. Researchers in the last decade have mostly focused on PFSSP, however, in common industrial systems, we have NPFSSP. Ample research is carried out on PFSSP, while research on the optimization of NPFSSP is very little and is still underdeveloped in the literature. The quality of results for NPFSSP is not very good as small instances are solved while the solution for large instances is still unsolved. The reason is not practical applications of the problems but rather simple permutation schedules of PFSSP. NPFSSP finds extensive applications within computer-integrated manufacturing, the chemical sector, and flexible manufacturing systems; nonetheless, there exists a limited number of solution methodologies for its practical implementation.

Research work on NPFSSP is limited as compared to the largely solved PFSSP, where the main reason is the hardness of NPFSSP. In PFSSP, an optimal solution is searched from (*n! )* feasible solutions, where “n” is the number of jobs. In NPFSSP the optimal solution is searched from (*n! )*^*m*^ feasible solutions, where “m” is the number of machines. Hence, the search space for NPFSSP is much larger as compared to PFSSP. The solution obtained from NPFSSP should be equal to or better than the solution found using PFSSP. However, with the rise of powerful computers, interest in solving NFPSSP has increased and is currently a favored research area in flow shop scheduling literature. The set of solutions for the PFSSP constitutes a subset of the solutions for the NPFSSP^2^. The term “completion time” denotes the finishing time of the final job on the last machine, which is also referred to as makespan; this metric is the primary objective examined in the optimization of PFSSP^3^.

The most common objective for NFPSSP is the minimization of makespan^2^. Other objectives frequently studied in NPFSSP are: minimizing weighted mean tardiness^4^, minimizing total tardiness^5^, minimizing weighted mean completion time^4^, and total flow time^6^, among others. In this paper, a HES algorithm is proposed for the minimization of makespan for NPFSSP and is tested on Demirkol, et al.^7^ benchmark problems. The initial solution is produced through the NEH heuristic, followed by the application of an IES algorithm to enhance the solution further. To avoid the risk of becoming ensnared in a local minimum and to investigate superior solution regions, a Local search technique is integrated into the IES algorithm. To save computational time and explore more solution space, the Quad swap mutation operator is used in IES. To avoid cyclic repetition of the same chromosome, a frequency table is used and each chromosome can be mutated a maximum of 50 times.

In “Literature Review” Section 2, a comprehensive literature review is presented, offering an analysis of the NEH heuristic and the ES algorithm. “Problem Statement” Section succinctly outlines the problem statement along with its associated constraints, whereas “Methodology” Section provides a detailed discussion of the methodology employed. “Computational Results” Section focuses on the presentation of computational results. Lastly, "Conclusion and Recommendations" Section concludes with a discussion of findings and recommendations.

## Literature review

Garey, et al.^8^ studied the complexities of FSSP and classified them as NP-complete problems for more than two machines. Hence solving them through exact methods is infeasible as they take ample computational time. Solution techniques for FSSP can be categorized as exact and heuristic methods. Exact methods can solve small-sized problems however they are not adequate to solve medium and large-size problems, moreover, they require ample computational time. Hence heuristic approaches are suitable to solve complex problems. The heuristic approach can be further categorized as Constructive heuristics and Meta-heuristics^9^. Constructive heuristics^10,11^) are good at finding feasible solutions however for large-size problems it is inferior to the optimal solution. In addition, the constructive heuristic cannot be applied to general cases as they are problem-specific. Meta-heuristics find a near-optimal solution with significant experimental time and are recommended for combinatorial optimization problems. Hence, researchers have used various Meta-heuristics i.e. Genetic Algorithm (GA), Tabu Search (TS), Evolution Strategies (ES), Simulated Annealing (SA), Evolutionary Programming (EP), Ant Colony Optimization (ACO), and Swarm Intelligence, among others, to solve complex problems.

Rossit, et al.^2^ carried out a comprehensive survey of various techniques used to solve NPFSSP with various objective functions. He analyzed 72 papers on NPFSSP with a period spread from 1988 to 2016. He showed that more than 65% of papers on NPFSSP were written after 2007, which shows that it is one of the focused researched topics in flow shop problems right now. He also summarized various techniques used to solve NPFSSP and showed that the percentage contribution of Exact, Constructive heuristics, and Meta-heuristics in solving NPFSSP are 22%, 28%, and 44% respectively. Hence, it is clear that Meta-heuristics are mostly used to solve NPFSSP as they provide better results, and can solve all size problems. The author showed that schedules for NPFSSP yield a better solution than PFSSP as the former includes all the solutions of PFSSP; however, more computational time is required to solve NPFSSP. Recent studies in Meta Heuristics indicate that there are occasions when the algorithm becomes ensnared in local minima, making it exceedingly challenging for the algorithm to escape from these local minima. Recently ample research has been performed on Hybrid Meta-heuristics in which different methods are combined to take advantage of each method. The optimal Hybrid Meta-heuristic will integrate the exploration and exploitation capabilities of diverse methodologies to yield the most effective outcomes.

The most prevalent objective function employed in addressing the FSSP is the minimization of makespan. Most of the researchers use makespan as a single objective function^2^. Janiak^12^ was the first researcher to minimize the makespan of NPFSSP; his algorithm was based on the Branch and Bound (B&B) Method and Disjunctive Graph Theory. A detailed performance evaluation of FSSP was carried out by Liao, et al.^13^ for Permutation vs. Non-Permutation schedules. The initial solution was found using three heuristics and was improved using the TS algorithm. Based on completion time-based criteria, the Permutation Schedules (PS) can be improved using Non-Permutation Schedules (NPS), however, the percentage improvement is rather small. While using the Tardiness and Weighted tardiness criteria, the percentage improvement is more specifically for more than 30 jobs. The experimental findings indicated that NPS outperformed PS in terms of performance. Haq, et al.^14^ proposed a Scatter Search (SS) approach for NPFSSP and tested its technique on Demirkol benchmark problems. The algorithm had a unifying principle that avoids repetition of the solution by using the adaptive memory principle.

For NPFSSP, Ying and Lin^15^ suggested a Multi Heuristic Desirability Ant Colony System. With makespan as the objective, the algorithm was tested on Demirkol instances. Lin and Ying^9^ proposed a Hybrid approach for NPFSSP to minimize makespan using SA and TS algorithms. The author found new upper bounds for 40 instances of Demirkol. A Hybrid Novel Quantum Differential Evolution Algorithm (QDEA) was suggested by Zheng and Yamashiro^16^ for NPFSSP, the Hybrid algorithm combined Local search, Differential operations, and Q-bit search. Ying^17^ proposed an Iterated greedy (IG) algorithm for NPFSSP and validated his results on Demirkol benchmark problems. Rossi and Lanzetta^18^ suggested a Native Non-Permutation algorithm using the ACO algorithm for NPFSSP. To build a native solution he used a digraph approach and validated his technique on Demirkol benchmark problems. Ziaee and Karimi^19^ developed a Mixed Integer Programming (MIP) model to minimize the Total Tardiness (TT) of NPFSSP. Preemption-dependent processing times and job due dates are used to minimize the TT. The algorithm underwent evaluation using instances that were generated at random.

For non-availability intervals in NPFSSP, a Hybrid Incremental Genetic algorithm (HIGA) was proposed by Cui, et al.^20^. The author investigated two types of intervals, in which the former intervals are fixed and known in advance while later intervals are flexible and cannot exceed a threshold time. For large-size problems, the Hybrid algorithm combines an incremental GA algorithm, a population diversity supervision scheme, and a local refinement scheme. With makespan as the objective, Ye, et al.^21^ solved NPFSSP having time lag constraints. The preliminary solution was developed through the application of a PFSSP heuristic, followed by the utilization of an IG algorithm to pinpoint high-quality non-permutation schedules. Benavides and Ritt^22^ suggested two heuristics for NPFSSP minimizing their makespan, the two heuristics used were Constructive and an Iterated Local Search (ILS) heuristic. By utilizing a few local inversions in both the heuristics, permutation structure is observed in optimal non-permutation schedules. Using unavailability constraints, Assia, et al.^23^ Minimized Total Energy Consumption (TEC) of NPFSSP, investigated two unavailability constraints using Mixed Binary Integer Programming (MBIP) model.

Benavides and Ritt^24^ employed Taillard’s acceleration technique to develop three heuristics for NPFSSP. IG algorithm combined the three heuristics i.e. a Constructive heuristic, a Non-Permutation Insertion Local Search, and a Reduced Neighborhood Best Improvement Local Search to minimize makespan. In the context of retail order picking within the NPFSSP framework, characterized by absent operations, transportation delays, and restricted capacity limitations, Souiden, et al.^25^ introduced a mathematical model aimed at reducing the overall time taken by all machines involved. With time couplings and makespan objectives, Idzikowski, et al.^26^ used three methods i.e. B&B method, TS, and GA algorithm to solve them and tested the results on Taillard (Taillard^27^) problems. With makespan as the objective function and stochastic processing times, Rossit, et al.^28^ introduced a Novel Approach for the combinatorial analysis of FSSP with two job cases. The goal was to determine the dominance properties between PFSSP and NPFSSP. Brum, et al.^29^ reduced the overall completion time (∑C) in the NPFSSP by proposing a framework for the development of IG algorithms and implementing an automatic algorithm configuration to achieve more efficient outcomes. A high-quality permutation schedule is first created, which is subsequently transformed into a non-permutation schedule by altering the sequence of jobs on select machines. The outcomes were verified using benchmark problems from Taillard and VRF (Vallada, et al.^30^).

Over the years researchers have mostly focused on GA for scheduling problems as they are powerful and can solve complex problems which are difficult to be solved using conventional techniques. GA has been successfully used in industrial engineering, operations research, management science, system engineering, etc. However, GA requires ample computational time, and sometimes it gets trapped in local minima^31^. To overcome these difficulties, the ES algorithm is used in this research. ES is a stochastic search heuristic and is a specialization of Evolutionary algorithms used for solving various optimization problems. ES mimics adaptive procedures in biological evolution. ES is mostly applied to continuous black-box optimization problems. The main operators of the ES are initialization, recombination, mutation, evaluation, and selection. In the initialization phase, an individual solution is generated and its fitness is evaluated. The evolutionary loop begins after the initialization phase and continues through recombination, mutation, evaluation, and selection until the termination requirements are met. Mutation and recombination are the main operators in ES to create offspring and have equal importance in the performance of the algorithm. Based on the objective function value, the selection operator selects individuals who are transferred to the next iteration.

The basic types of ES are Two membered-ES and Multi membered-ES. In a Two-membered, one offspring is generated from a parent. In multi-membered-ES, more than one offspring is generated from a single parent. One unique characteristic of ES that distinguishes it apart from GA, which has a constant mutation rate, is the mutation operator’s self-adaptation. The mutation operator signifies the magnitude and direction of changes in the origin of the new position of the individuals in the search space. ES is commonly represented as (µ + λ)-ES, where µ represents the number of parents and λ represents the number of offspring. For recombination operators, different values of λ can be used i.e. 4, 8, 9, and 16 (Paris, et al.^32^). It is advised to choose λ = 4 for the minimal search of solution space with the least amount of computational time and λ = 16 for the highest search of solution space at the expense of computational time. Although ES is commonly applied to a continuous optimization problem, however by modifying the recombination and mutation operator was successfully applied to the discrete optimization problem by Cai and Thierauf [65]. For continuous optimization problems, there should be minor variations in all the components of a parent [40]. Small variations in parents reflect small mutations, which mimic natural biological evolution. In discrete optimization problems, constituents of a parent take their value from a discrete set, so a random variation arises from one discreet value to another nearby discrete value. Normally there is a large difference between two adjacent values, hence varying all components of a parent will result in a high mutation in the objective function. For better results, Cai and Thierauf [65] advised altering a few components in a parent. For more detail, on ES the readers should refer to [66], [67].

To date application of ES in the scheduling field is very limited, although it has been successfully applied to various optimization problems i.e. Image Filtering^32^, Capacitated Vehicle Routing Problem^33^, Structural Shape Optimization^34^, Designing Of Metal Forming Die Surfaces^35^, Feedforward And Recurrent Networks^36^, Multiprocessor Scheduling^37^, Mobile Manipulator Path Planning^38^, Multigrid Problems^39^, Wireless Sensor Networks^40^, Automatic Berthing^41^ and Electricity Load Forecasting Problems^42,43^). For Hybrid Flexible Flow Shop Problems, de Siqueira, et al.^44^ Proposed an algorithm based on the ES algorithm. The initial solution was generated using the NEH heuristic and IG algorithm. A Hybrid GA algorithm was proposed by Zhang, et al.^45^ by combining ES, Local search, and Population Diversity Supervision Scheme for Periodical Maintenance (PM) of FSSP. Khurshid, et al.^46^ proposed a Hybrid ES algorithm for Robust PFSSP, in which he utilized the global search abilities of ES and combined it with TS to exploit its Local search abilities. For the recombination operator, λ = 9 is used and for the mutation operator, double quad swap mutation is used. Two Fast Evolutionary algorithms based on ES were proposed by Khurshid, et al.^47^ to minimize the makespan of PFSSP. Results of the proposed algorithm were also validated in a real-life case of battery manufacturing, and the results show that by using ES the company can significantly increase its production. Khurshid, et al.^48^ combined an Improved ES algorithm with SA to minimize the makespan of PFSSP and the results were validated on Taillard benchmark problems. ES was also utilized to solve BFSSP with makespan as the objective function^49^ In another research on makespan minimization in the NWFSSP, Khurshid, et al.^50^ combined the Iterated Greedy (IG) algorithm with the ES algorithm. IG algorithm is famous for its simplicity in solving FSSP and the hybridization with ES leads to a robust algorithm that efficiently solved benchmark Taillard and Carlier problems. Recently ES was extended to Job Shop Scheduling Problems by Khurshid and Maqsood^51^ with minimization of makespan as the objective function. Together with SA, the ES creates the initial solution at random, enhancing the hybrid algorithm’s local search capabilities and preventing it from becoming stuck in local minima. Table 1, compares ES with other techniques in the literature used to solve NPFSSP.

**Table 1:** Comparison of techniques to solve NPFSSP.

Author	NPFSSP	Technique	Performance Measures				Problem Set
			No of machines	Objective	Quad Swap Operator	Frequency Table	
Janiak ^12^	✓	B&B	m	C_max_			
Demirkol, et al. ^7^	✓	SB	m	C_max_			Demirkol
Haq, et al. ^14^	✓	SS	m	C_max_			Demirkol
Ying and Lin ^15^	✓	H	m	C_max_			Demirkol
Lin and Ying ^9^	✓	SA-TS	m	C_max_			Demirkol
Zheng and Yamashiro ^16^	✓	QDEA	m	C_max_			Demirkol
Ying ^17^	✓	IG	m	C_max_			Demirkol
Rossi and Lanzetta ^18^	✓	ACO	m	C_max_			Demirkol
Zhang et al. ^45^		HES		PM			
Benavides and Ritt ^22^	✓	H	m	C_max_			
Ziaee and Karimi ^19^	✓	IP	m	TT			
Cui, et al. ^20^	✓	HIGA	m	C_max_			Demirkol
Ye, et al. ^21^	✓	H	m	C_max_			
Assia, et al. ^23^	✓	MBIP	m	TEC			
Benavides and Ritt ^24^	✓	H	m	C_max_			Taillard
Brum, et al. ^29^	✓	IG	m	∑C			Taillard, VRF
Khurshid, et al. ^46^		HES	m	C_max_			Carlier and Reeves
Khurshid, et al. ^47^		ES	m	C_max_	✓		Carlier and Reeves
Khurshid and Maqsood ^51^		HES	m	C_max_			Taillard
This paper	✓	HES	m	C_max_	✓	✓	Demirkol

## Problem Statement

In NPFSSP, *n*-jobs are processed on *m*-machines. A job represents an individual task or work unit that must be processed across multiple machines in a specific order to complete. The processing sequence refers to the specific order in which each job will visit the machines to complete its required operations. Each job is processed by m operations with an order *1*,*…*,*m*. The *i*^*th*^ operation of a job is processed by the *i*^*th*^ machine. The sequence of operation for machines can be different. Preemption is prohibited, and a job once loaded on a machine will be processed uninterrupted for the entire processing time. Processing times on the machine (including setup times) are known in advance and the number of jobs is known. All machines are thoroughly available and the number of machines is known. At time zero, any job can be started first. The storage capacity of buffers is unlimited.

P_ij_ is the processing time of job *j* on machine *i*. The processing of the job is completed once it finishes visiting all the machines. Let *C*_*ij*_ be the completion time of job *j* on machine *i*. The total completion time also termed makespan can be calculated as follows:

$$\:{C}_{max}=\sum\:_{i=1}^{n}{C}_{mj}\:\:\:\:\:\:\:\:\:\:\:\:\:\:\:\:\:\:\:\:\:\:\:\:\:\:\:\:\:\:\:\:\:\:\:\:\:\:\:\:\:\:\:\:\:\:\:\:\:\:\:\:\:\:\:\:\:\:\:\:\:\:\:\:\:\:\:\:\:\:\:\:\:\:\:\:\:\:\:\:\:\:\:\:\:\:\:\:\:\:\:\:\:\:\:\:\:\:\:\:\:\:\:\:\:\:\:\:\:\:\:\:\:\:\:\:\:\:\:\:$$

Examine the following variables along with a large positive constant M.

$$\:{Z}_{\text{i}\text{j}\text{k}}=\left\{\begin{array}{cc}1&\:\text{i}\text{f}\:\text{j}\text{o}\text{b}\:j\:\text{i}\text{s}\:\text{s}\text{c}\text{h}\text{e}\text{d}\text{u}\text{l}\text{e}\text{d}\:\text{b}\text{e}\text{f}\text{o}\text{r}\text{e}\:\text{j}\text{o}\text{b}\:k\:\text{o}\text{n}\:\text{m}\text{a}\text{c}\text{h}\text{i}\text{n}\text{e}\:i\\\:0&\:\text{o}\text{t}\text{h}\text{e}\text{r}\text{w}\text{i}\text{s}\text{e}\end{array}\right.\:\:\:\:\:\:\:\:\:\:\:\:\:\:\:\:\:\:\:\:\:\:\:\:\:\:\:\:\:\:\:\:\:\:\:\:\:\:\:\:\:\:$$

$$\:{s}_{ij}=\text{s}\text{t}\text{a}\text{r}\text{t}\text{i}\text{n}\text{g}\:\text{t}\text{i}\text{m}\text{e}\:\text{o}\text{f}\:\text{j}\text{o}\text{b}\:j\:\text{o}\text{n}\:\text{m}\text{a}\text{c}\text{h}\text{i}\text{n}\text{e}\:i\:\:\:\:\:\:\:\:\:\:\:\:\:\:\:\:\:\:\:\:\:\:\:\:\:\:\:\:\:\:\:\:\:\:\:\:\:\:\:\:\:\:\:\:\:\:\:\:\:\:\:\:\:\:\:\:\:\:\:\:\:\:\:\:\:\:\:\:\:\:\:\:\:$$

The mathematical model to calculate the makespan of NPFSSP is as follows.

1 $$Minimize:\:\:\:\sum\:_{j\in\:n}\left({s}_{mj}+{p}_{mj}\right)$$

s.t

2 $$\:M{Z}_{ijk}+\left({s}_{ij}-{s}_{ik}\right)\:\ge\:\:\:{p}_{ik}\:\:\:\text{i}=1,\dots\:,\text{m};\:\:\:\:1\le\:\text{j}\le\:\text{k}\le\:\text{n}\:$$

3 $$\:M(1-{Z}_{ijk})+\left({s}_{ik}-{s}_{ij}\right)\:\ge\:\:\:{p}_{ij}\:\:\:\text{i}=1,\dots\:,\text{m};\:\:\:\:1\le\:\text{j}\le\:\text{k}\le\:\text{n}$$

4 $$\:{\text{s}}_{ij}+\:{p}_{ij}\:\le\:{\text{s}}_{i+1,j}\:\:\text{i}=1,\dots\:,\text{m};\:\:\:\:\text{j}=1,\dots\:\text{n}\:\:$$

5 $$\:{\text{s}}_{ij}\ge\:0,\:{Z}_{ijk}\in\:\left\{\text{0,1}\right\}\:\:\:\:\text{i}=1,\dots\:,\text{m};\:\:\:\:\text{j},\text{k}=1,\dots\:\text{n}\:$$

Equations 2, and 3 ensure that one job is processed on one machine only. Equation 4 ensures that a job is finished on a machine before it is loaded on the next machine. Equation 5 defines the domains of the variables.

## Methodology

### NEH Heuristic

In FSSP for the minimization of makespan, the NEH heuristic^10^ is regarded as the best constructive heuristic of all times. Ruiz and Maroto^52^ compared various heuristics available for PFSSP and has shown that the NEH heuristic achieves best results for makespan objective function. The superiority of the NEH heuristic over other heuristics was also investigated by Kalczynski and Kamburowski^53^. To obtain further information and practical uses of the NEH heuristic, the readers should consult the papers of Kalczynski and Kamburowski^54^, Dong, et al.^55^, and Kalczynski and Kamburowski^56^. The NEH heuristic works in three steps. It initially sequences the job by the sum of completion times in descending order on all the machines. In the second step, two jobs with the maximum value of work content are selected first and their makespan is calculated. In step three, the remaining jobs are then placed at possible positions so that the makespan is minimized. Job 3 ≤ *i* ≤ *n* can be inserted into the partial sequence in *i* different positions. Using Taillard acceleration, the NEH algorithm’s computational time was lowered from O(n^3^ m) to O(n^2^ m)^11^. Combining the NEH heuristic with other Meta-Heuristics yields the best results as claimed by numerous researchers. Stützle^57^ combined the NEH heuristic with the ILS algorithm, Tabu search was combined with NEH heuristic by Grabowski and Wodecki^58^, the NEH heuristic was combined with two GA by Ruiz, et al.^59^, Ruiz and Stützle^60^ combined Iterated Greedy algorithm with NEH heuristic, Whale optimization algorithm combined NEH heuristic by Abdel-Basset, et al.^61^, and Aqil^62,63^ combined NEH heuristic with population based meta-heuristics i.e. IG algorithm, Migratory Bird Optimization, Artificial Bee Colony algorithm, and ILS algorithm.

**Fig. 1:**
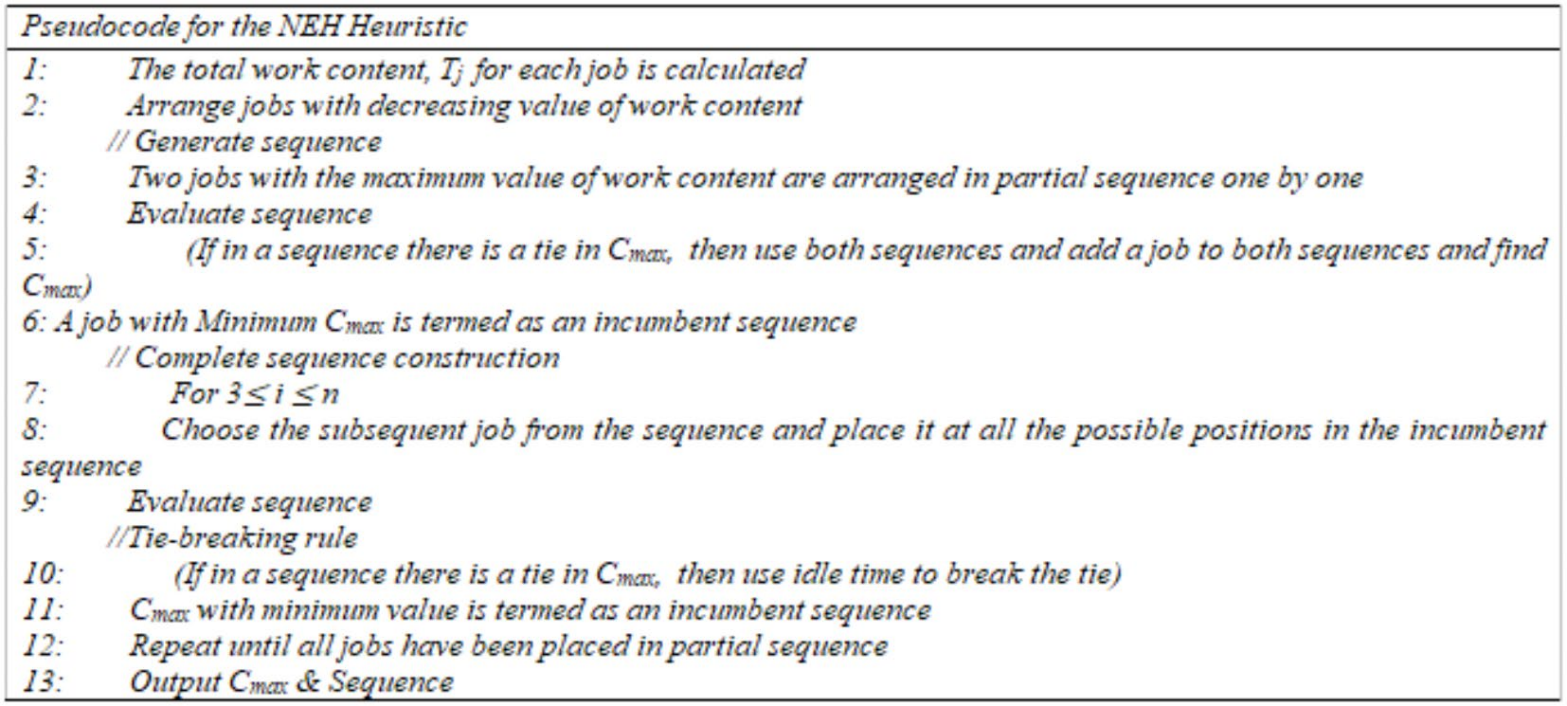
Pseudo Code for NEH Heuristic.

The main strength of the NEH heuristic is the initial arrangement of jobs and jobs insertion phase, while its weakness is the large number of ties during step 3. The tie-breaking mechanism of Fernandez-Viagas and Framinan^64^ which is based on minimum idle time has shown very good performance as it does not increase the O(n^2^ m) complexity of the NEH heuristic. Figure 1 displays the pseudocode for the NEH heuristic. Over the past 20 years, various Meta-heuristics^58,65,58,66,67^) have been used to solve PFSSP with the minimization of makespan as the objective function. In most of these Meta-heuristics, the initial sequence was found using the NEH heuristic. Local search methods i.e. GA, SA, ACO, and TS, among others, were used to improve the sequence, and these local search methods found very good results when combined with the NEH heuristic^53^. Hence, the NEH heuristic is also used to generate the initial sequence in this paper. The proposed HES works in two phases, in the initial phase solution for the NPFSSP is found using the NEH heuristic (a permutation schedule is generated). The outcomes of the NEH heuristic are optimized in the second phase using the IES method. The IES algorithm work in two steps, for first 1000 iterations the permutation schedule found from NEH is further improved while in the next 4000 iterations, a non-permutation schedule is improved using the IES algorithm. Therefore, the IES Algorithm uses the NEH Heuristic solution as a seed.

### Evolution strategies (ES)

Darwinian Theory of Evolution i.e. Stronger tends to survive while weaker tend to die is the basis for Evolutionary algorithms. The subclass of Evolutionary Algorithms is Evolutionary Programming, ES, GA, and Genetic Programming. Conferring to Darwin’s theory, the principles of variation and selection are the fundamental principles in the development of species. All variants of Evolutionary algorithms have the same essence: offspring are generated from a parent population and then the best offspring are selected to generate a new parent for the next iteration. ES was developed by Rechenberg^68^ in 1970 and is a stochastic optimization algorithm. They were further improved by Schwefel^69^. The first application of ES was to design optimal shapes for the win shape and nozzle using physical experiments. High-performing structures with astonishing shapes were discovered due to the evolutionary design of ES. Originating from hill climbing strategies, ES was applied to other complex problems such as black optimization problems by using high-performance computers. The introduction of mutation distribution of adaptive step sizes contributed to the success of ES. Although some stochastic search algorithms utilized step-size adaptation, a flexible adaptation scheme in mutation distribution was focused on by researchers to improve its performance. This feature of ES distinguishes it from a GA where a constant mutation rate is used. For the adaptation of mutation parameters, three mainstream variants were developed. First, control a single step size by using the 1/5th success rule. Second, mutation self-adaptation which mimics natural evolution, and third, efficient de-randomized self-adaptation scheme. Attempts to find an effective de-randomized adaptation scheme let Hansen, et al.^70^ discover covariance matrix adaptation-ES, which is highly suitable for global optimization problems.

Population-based-ES also known as multi-membered-ES was developed in parallel and performed as collective Hill-climbing termed by Schwefel^71^. Multi-membered-ES is more suitable for noisy and global optimization problems, as they exploit the affirmative effects of recombination operators. Population-based-ES can be run in parallel and can be extended to advance ES for solving multi-modal and multi-objective optimization tasks. Presently, ES is mostly used to solve Simulation-based optimization. As ES does not require derivatives, hence it is also applicable to solve the optimization of non-smooth functions. Main variants of ES are used for continuous optimization problems, unlike GA which is suited for binary search spaces. Modern-ES are as efficient as other derivative-free optimization algorithms and are successfully applied to a large number of system optimization and engineering problems^72^. In ES, the whole population is simultaneously processed, unlike other algorithms where few solutions are processed from the population. The primary operators for ES are, recombination and mutation to produce offspring. ES is self-adaptable, meaning it can vary some parameters during a run. Initially, Hartmann^73^ used ES for numerical applications, and later Schwefel^69^ used it to solve discrete and binary parameter optimization problems. The key features of ES are as follows.

- They are recommended for real value optimization problems.
- The main source of genetic variation is a mutation, unlike GA where the crossover is the main genetic operator.
- Crossover is not used and offspring are randomly generated from a parent by recombination.
- A deterministic procedure is used for selection.
- The mutation rate is varied during iterations to achieve faster results as mutation operators are parameterized.

ES is defined by the notion (µ + λ)-ES, where µ represents the number of parents and λ represents the number of offspring. ES can be classified into two types, Two membered-ES, and Multi membered-ES. The Two membered-ES is also represented as (1 + 1)-ES which is simply a mutation scheme and the population consists of a single parent. Initial experiments were accomplished using one parent and one offspring and mutation was carried out by withdrawing two numbers from a normal distribution. The parent was replaced by its offspring if it was better. With the invention of computers and the introduction of the population concept by Rechenberg, the Two membered (1 + 1)-ES was replaced with a Multi membered-ES. Additionally, the concept of recombination operators is incorporated in the Multi-membered-ES. Now, a parent produces λ offspring in one repetition. The Multimembered-ES consists of two basic stages. Step one uses both recombination and mutation to create offspring. However, a method for choosing survivors is used in the second stage. Recombination operators can be classified into intermediate and discrete operators. In the former category, parental variable values are selected from parents at random. While in the later category, the offspring gets the average value of its parents. Both mating selection and the need that the parent involved to be distinct are absent. For continuous variables, 100% mutation and 100% recombination are carried out. If the parents take part in the selection process it is termed as (µ + λ)-ES otherwise it is termed as (µ, λ)-ES where parents die out of the selection phase and only offspring take part in the selection process. Because only the finest parents are kept and the rest are forgotten, the selection process is referred to as truncation selection. It is advised for recombination operators to use different values of λ. Paris, et al.^32^ Used λ = 4, 8, and 16 in their research on image processing.

**Fig. 2:**
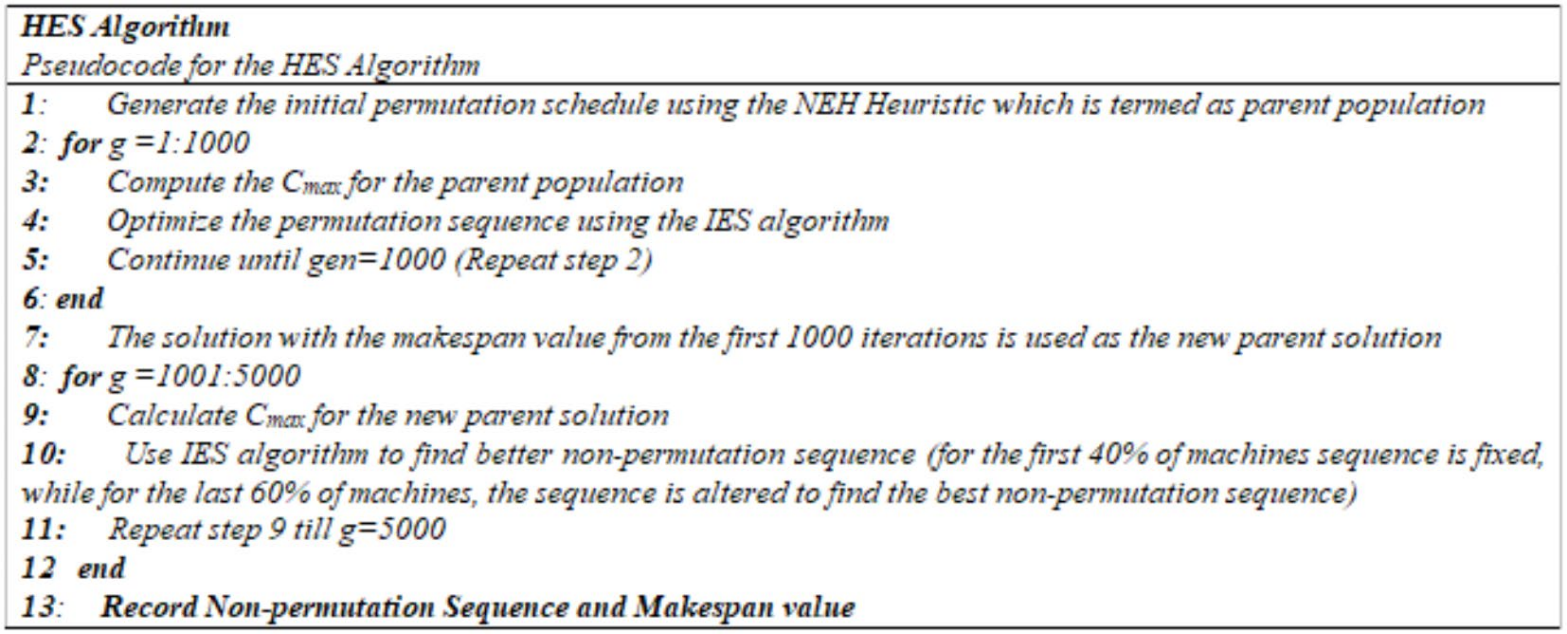
Pseudo Code for HES algorithm.

To locate a better nearby solution and avoid local minima, the ES method should be paired with any local search technique as it occasionally becomes caught in one. In the proposed research, the exploitation ability of the NEH heuristic is combined with the exploration ability of IES for optimum results with minimum computational time. To find better solutions and escape local minima, the IES algorithm is equipped with a Local Search Technique. Rossit, et al.^2^ showed that the possible solution of PFSSP is a subset of NPFSSP. Therefore, to find a good non-permutation schedule, first, start with a better permutation schedule and then modify it to find a good non-permutation schedule. So in this research, first a permutation schedule is generated using the NEH heuristic. The permutation schedule acts as a parent solution for the IES algorithm, which further improves the permutation schedule for the next 1000 iterations. Then a non-permutation schedule is generated and improved using the IES algorithm in the next 4000 iterations. Figure 2 shows the Pseudo code of HES, which shows the generation scheme of the Permutation and Non-permutation schedule using the NEH heuristic and IES algorithm respectively. In step 10, for finding a non-permutation sequence, the permutation sequence found is fixed on the first 40% machines and is not changed for the next 4000 iterations, i.e. The sequence for the first 40% machines is the same as found from the previous 1000 iterations. For the last 60% of machines, the non-permutation sequence is obtained by applying the IES algorithm and the sequence is varied so that the makespan is minimized. Therefore, in the second stage of the IES algorithm, a non-permutation sequence is obtained by altering the sequence of the last 60% of machines only. As shown in Fig. 1, for the first two machines permutation sequence is fixed, while in the last two machines, the sequence is varied so that the makespan is minimal as compared to the makespan found using the permutation sequence.

### Improved evolution strategies (IES)

To enhance the performance of (1 + 1)-ES, and to ensure simultaneous intactness of exploitation and exploration ability of the ES algorithm, the following improvements are suggested.

- To explore the solution space as much as possible in the shortest amount of time, utilize the quad-swap mutation operator.
- For recombination, λ = 16 is used, hence a parent solution generates sixteen offspring at random.
- For selection, (µ + λ) is used in this paper, hence both the offspring and parent are involved in the selection process. If the makespan of a parent solution is superior to that of the generated offspring, the parent solution may persist through multiple iterations, unless it is replaced by a more optimal offspring.
- To guide the search to unexplored areas and increase exploration, a frequency table is used. In the frequency table, a record of the mutated chromosome is maintained and a chromosome can be mutated a maximum of 50 times.
- To escape local minima, a Local Search Technique is utilized.
- IES is applied for the optimization of Demirkol Benchmark problems for the very first time.

**Fig. 3:**
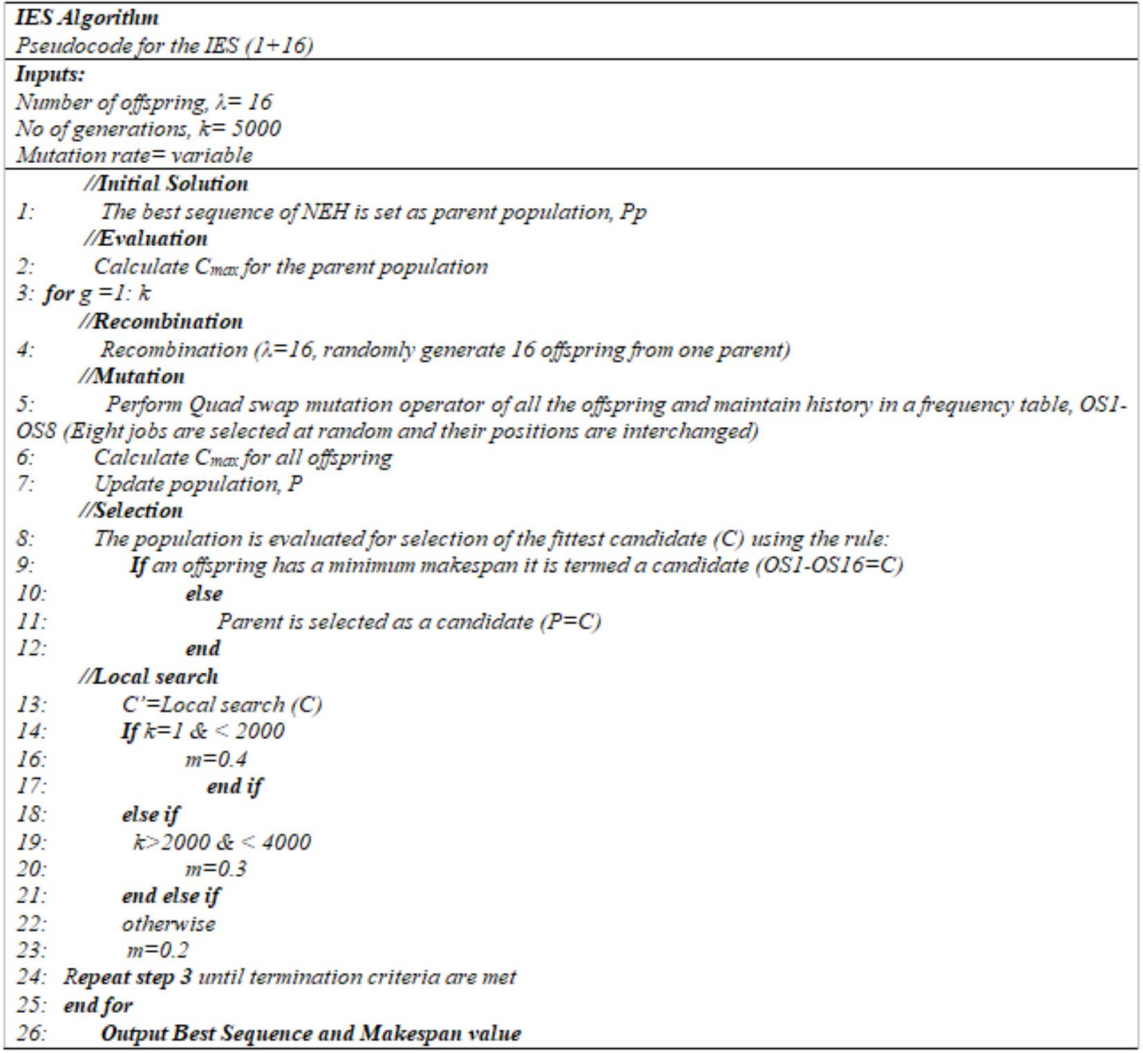
Pseudo Code for IES algorithm.

Figure 3 shows the Pseudo Code for Improved ES algorithm.

#### Initialization

The *a* individuals build a parent population from *µ* parent *a*_*m*_, where *m = 1*,*…*,* µ* and *λ* offsprings *ã*_*l*_ where *l = 1*,*…*,* λ*. The parent and offspring population at iteration *g* are symbolized as $\:{{\beta\:}_{\mu\:}}^{k}$ and $\:{{\beta\:}_{\lambda\:}}^{k}$ where

1 $$\:{{\beta\:}_{\mu\:}}^{k}=\:\left\{{a}_{m}^{g}\right\}=\left({a}_{1}^{g},\dots\:,\:{a}_{\mu\:}^{g}\right)\:$$

2 $$\:{{\beta\:}_{\lambda\:}}^{k}=\:\left\{{a}_{l}^{g}\right\}=\left({a}_{1}^{g},\dots\:,\:{a}_{\lambda\:}^{g}\right)\:$$

3 $$\:{\beta\:}^{k}\to\:{\beta\:}^{k+1}$$

#### Selection

Based on the fitness values, the selection is used to find solutions before performing recombination. While fitness refers to the minimization of makespan during the exploration of solution space. Through a deterministic procedure, selection operators of type (+,) generate a parent population $\:{\beta\:}_{\mu\:}$ for the next iteration *g + 1*. According to fitness value F(y), the best individuals from a set of ***γ*** individuals *(a*_*1*_,*…*,*a*_*γ*_*)* are selected.

$$\:\left({a}_{1;\varvec{\upgamma\:},\:}{a}_{2;\varvec{\upgamma\:},\:\:}{\dots\:,a}_{\mu\:;\varvec{\upgamma\:},\:\:}\right)=selection\:({a}_{1},\dots\:,{a}_{\gamma\:})$$ where γ ≥ µ (4)

#### Recombination

In recombination, offspring are denoted as λ is generated from the parent which is denoted as µ. The main categories of recombination operators are intermediate and discrete recombination. In the former recombination, the properties of all the individuals are taken into account and the offspring takes their average properties. While in the latter recombination, individual parent properties are transferred to their offspring. In this paper λ = 16 is used, where sixteen offspring are produced from one parent at random. The recombination operator selects the parent ($\:\epsilon\:$) which takes part in the generation of offspring.

$$\:\epsilon\:=\left({a}_{1},\dots\:,{a}_{m},\dots\:,{a}_{\mu\:}\right)$$ and m = 1,…, µ (5)

The recombination operator itself provides little benefit, however, when it is combined with the mutation operator it provides useful results. Recombination guarantees that the offspring are similar to their parents.

#### Quad swap mutation operator

**Fig. 4:**
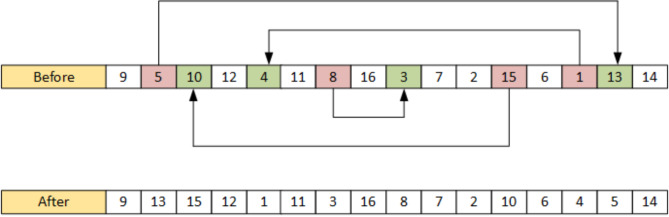
Quad swap mutation operator.

Mutation serves as the primary mechanism for generating genetic diversity within the ES algorithm. It introduces minor alterations at each iteration. A key advantage of the ES algorithm lies in its ability to adaptively modify the mutation operator. Given that the mutation operator is contingent upon the specific problem being addressed, the precise configuration of this operator significantly influences the overall efficacy of the ES. Notably, swap mutation is particularly effective for combinatorial optimization challenges. To maximize the exploration of the solution space efficiently, a Quad swap mutation operator is employed, which involves selecting four chromosomes and exchanging their positions, as illustrated in Fig. 4. The mutation rate is fixed during the iteration. The history of the mutated chromosomes is also maintained and each chromosome can be mutated a maximum of 50 times each, this guides the search of the algorithm to new promising areas. The new offspring ($\:{{\beta\:}_{\lambda\:}}^{k})$ are generated by randomly interchanging positions of parent population ($\:{{\:\beta\:}_{\mu\:}}^{k}$) at each iteration (k).

6 $$\:{{\beta\:}_{\lambda\:}}^{k}=random\left({{\beta\:}_{\mu\:}}^{k}\right)$$

7 $${\text{Randomly select any 8 jobs such that }}{{\text{a}}_{\text{1}}} \ne {{\text{a}}_{\text{n}}}{\text{and where n}}={\text{2}}:{\text{8}},$$

8 $${\text{Now swap }}{{\text{a}}_{\text{b}}} \leftrightarrow {{\text{a}}_{\text{c}}},{\text{ where b}}={\text{1}},{\text{2}},{\text{3}},{\text{4 and c}}={\text{8}},{\text{7}},{\text{6}},{\text{5}}$$

#### Frequency table

To avoid repetition of the same offspring, again and again, a frequency table is used. The frequency table stores the swapping moves of the mutated offspring. Maximum mutations for each chromosome are set at 50. Hence less mutated offspring are given priority in mutation, so the search is guided to less explored areas. The frequency table minimizes computational time and also increases the exploration ability of the IES algorithm.

#### Selection (survivor)

Based on fitness, the selection is used to deterministically choose the best µ-parents after creating λ-offsprings. The two selection operators available in ES are (µ + λ) and (µ, λ). In the former operator, the selection process involves both parents and the offspring, the best solution is termed as a parent for the next iteration. In the latter operator, parental individuals are eliminated from the selection process, allowing only their offspring to participate. The most optimal solution among the offspring is designated as the parent for the subsequent iteration. The (µ, λ) strategy is suggested for optimizing real-valued parameters. For combinatorial optimization (µ + λ) is recommended^75^ and is used in the paper.

#### Local search

**Fig. 5:**
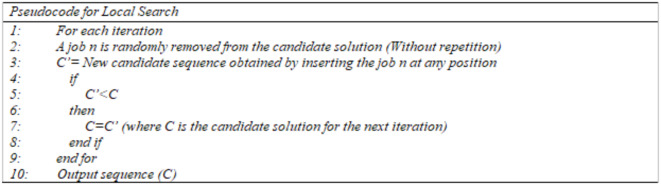
Local search procedure.

To guide the solution search to more promising areas and to avoid the local minima, a local search technique is incorporated in the IES algorithm. The insertion Local search technique of the IG algorithm of Ruiz and Stützle^60^ is used to generate new sequences and operate as follows. A job from a sequence is removed and inserted into another position. The process is repeated in each iteration to find a new solution that has a better makespan value. The history of the removed job is maintained so that repetition of the same move can be avoided and the search can be guided to less explored areas. Ruiz and Stützle^60^ showed that small modifications in the local search provide better solutions while large modifications cause a strong disruption. The pseudocode for the local search technique is shown in Fig. 5.

#### Termination

**Fig. 6:**
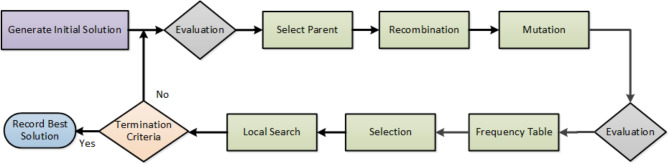
Mechanics of Hybrid Evolution Strategies.

Various termination criteria may be employed, such as computational time, upper bound values, lack of improvement in solutions over a designated number of iterations, and the total number of iterations, among others. In this study, the chosen termination criterion is the number of iterations, which is set at 5,000. Figure 6 illustrates the mechanics of the proposed Hybrid Evolution Strategy algorithm.

### Parameter optimization

The population size, selection operator, reproduction operator, mutation type, mutation rate, and termination criteria are the six main factors that make up the ES algorithm. Typically, the population size, determined by the reproduction operator, remains constant. As the population size increases, so does the computational time required for each iteration. For flow shop problems swap mutation operators perform better as compared to other mutation operators^74^. There are various types of swap mutation operators i.e. single swap mutation operators, double swap mutation operators, quad swap mutation operators, and k-swap mutation operators. For parameter optimization, the best-performing mutation operator is selected from single swap mutation operators, double swap mutation operators, and quad swap mutation operators as the k-swap mutation operator takes ample computational time. The mutation rate during the iteration is fixed. Different types of reproduction operators can be selected i.e. λ = 4, 5, 8, 9, and 16 ^32^. Various termination iterations, such as 3000, 3500, 4000, 4500, and 5000, can be employed. As a result, we need to figure out the ES algorithm’s three total parameters. The calibration of algorithm parameters is done using the Multifactor Analysis of Variance Design of Experiments^76^. The algorithm’s stopping criterion is 50xnxm milliseconds, where n and m are the number of jobs and machines, respectively. The algorithm is tested on eight instances i.e. DMU_20_15_10, DMU_20_20_10, DMU_30_15_9, DMU_30_20_10, DMU_40_15_10, DMU_40_20_9, DMU_50_15_8, DMU_50_20_8, and five iterations are performed for each instance to perform the parameter configuration.

The following factors are the focus of the computational experiment during the calibration phase: (I) the five levels of the reproduction operator (λ): 4, 5, 8, 9, and 16. (II) Mutation type at three levels: single swap mutation operators, double swap mutation operators, and quad swap mutation operators. (III) Five levels for termination criteria are used i.e. 3000, 3500, 4000, 4500, and 5000, resulting in 5 × 3 × 8 × 5 = 600 Average Relative Percentage Difference (ARPD) values.

In Table 2 results of a Multi-factor ANOVA are shown, the ARPD is the response variable, and it can be computed using Eq. (6).

6 $$\:ARPD=\frac{{C}^{*}-C}{C}$$

C* is the makespan of the ES algorithm, while C is the best makespan value for any instance.

**Table 2:** ANOVA results for the calibration phase.

Source	Sum of squares	Df	Mean square	*P*-value	F-ratio
Reproduction	0.32104	4	0.080260	0.000	16.23
Mutation type	0.00075	2	0.000376	0.927	0.08
Termination criteria	0.01139	4	0.002847	0.680	0.58

ANOVA involves finding the ideal factor concentrations to either maximize or decrease the response variable. Selecting the elements (independent variables) and their respective levels is the first step in designing an experiment^77^. After that, the design should let the elements be changed methodically to see how they affect the response variable (dependent variable). The following are the basic steps in an ANOVA: (1) Determine the average response for every factor level. (2) Determine the response’s overall mean. (3) Divide the overall variation into parts that can be attributed to every element and how they interact. (4) To calculate the F-statistic for each individual factor and their interactions, divide the mean square of each factor by the mean square of the error. Subsequently, to assess significance, compute the p-value corresponding to each F-statistic. A factor is considered significant if its p-value falls below the predetermined significance threshold, such as 0.05.

**Fig. 7:**
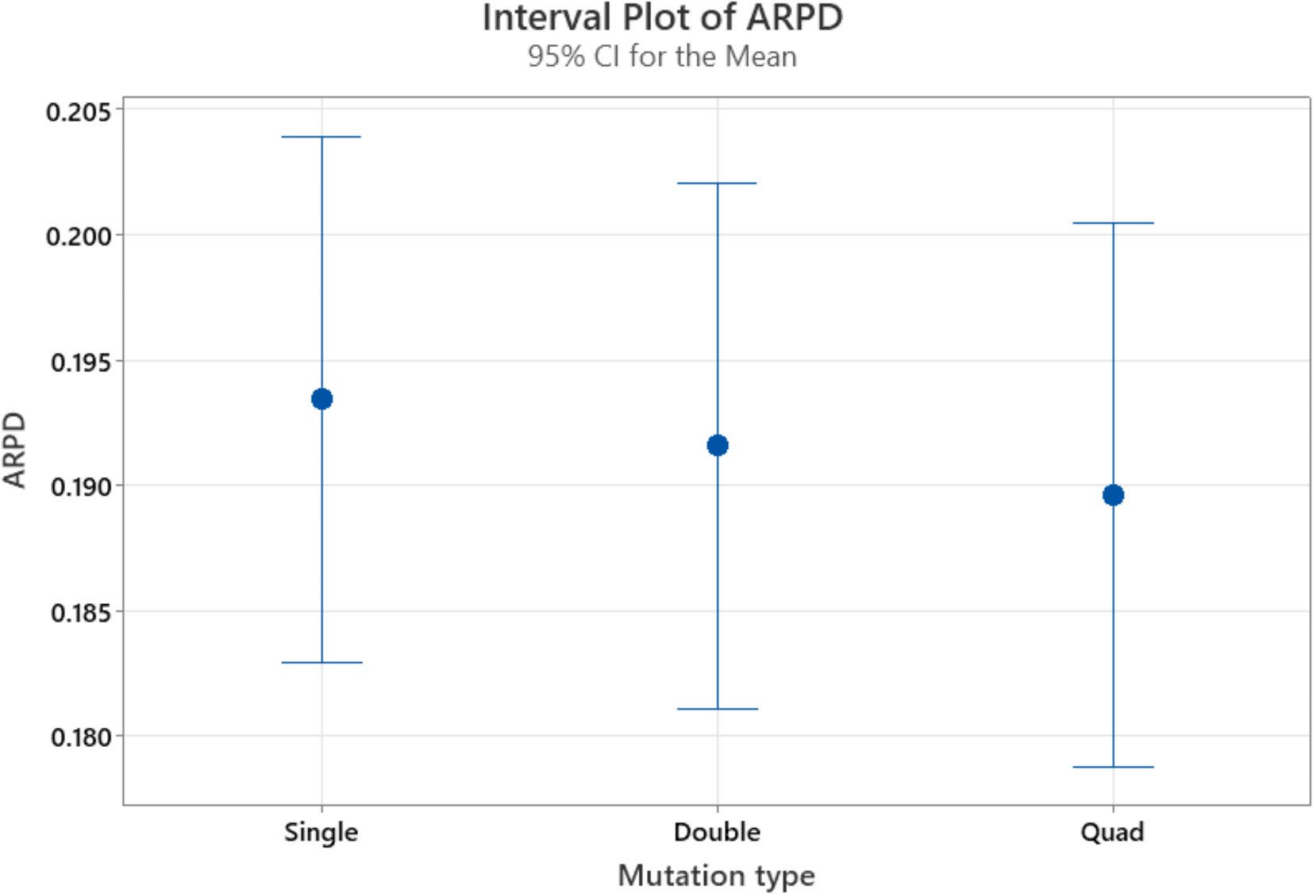
Box plot for mutation types.

**Fig. 8:**
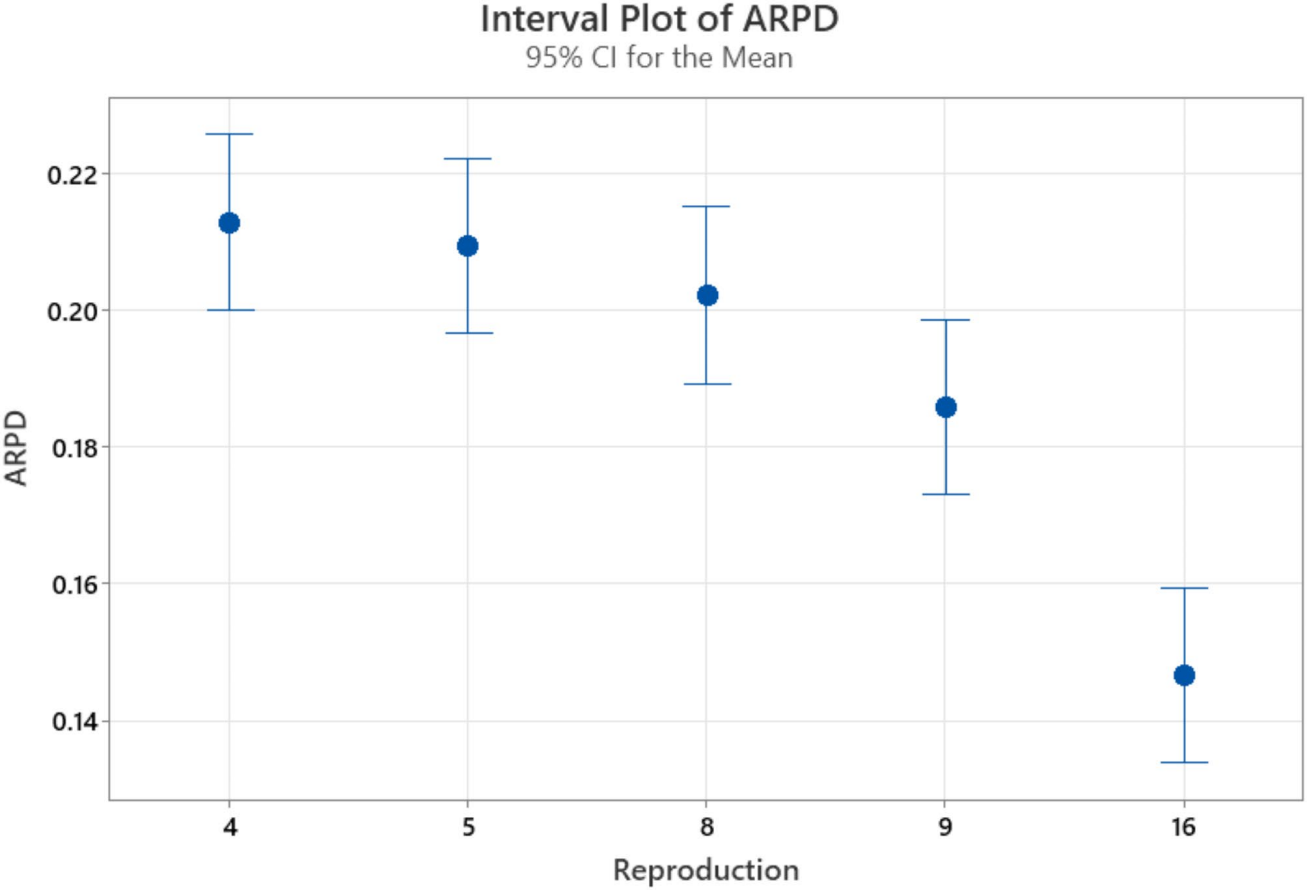
Box plot for reproduction types.

**Fig. 9:**
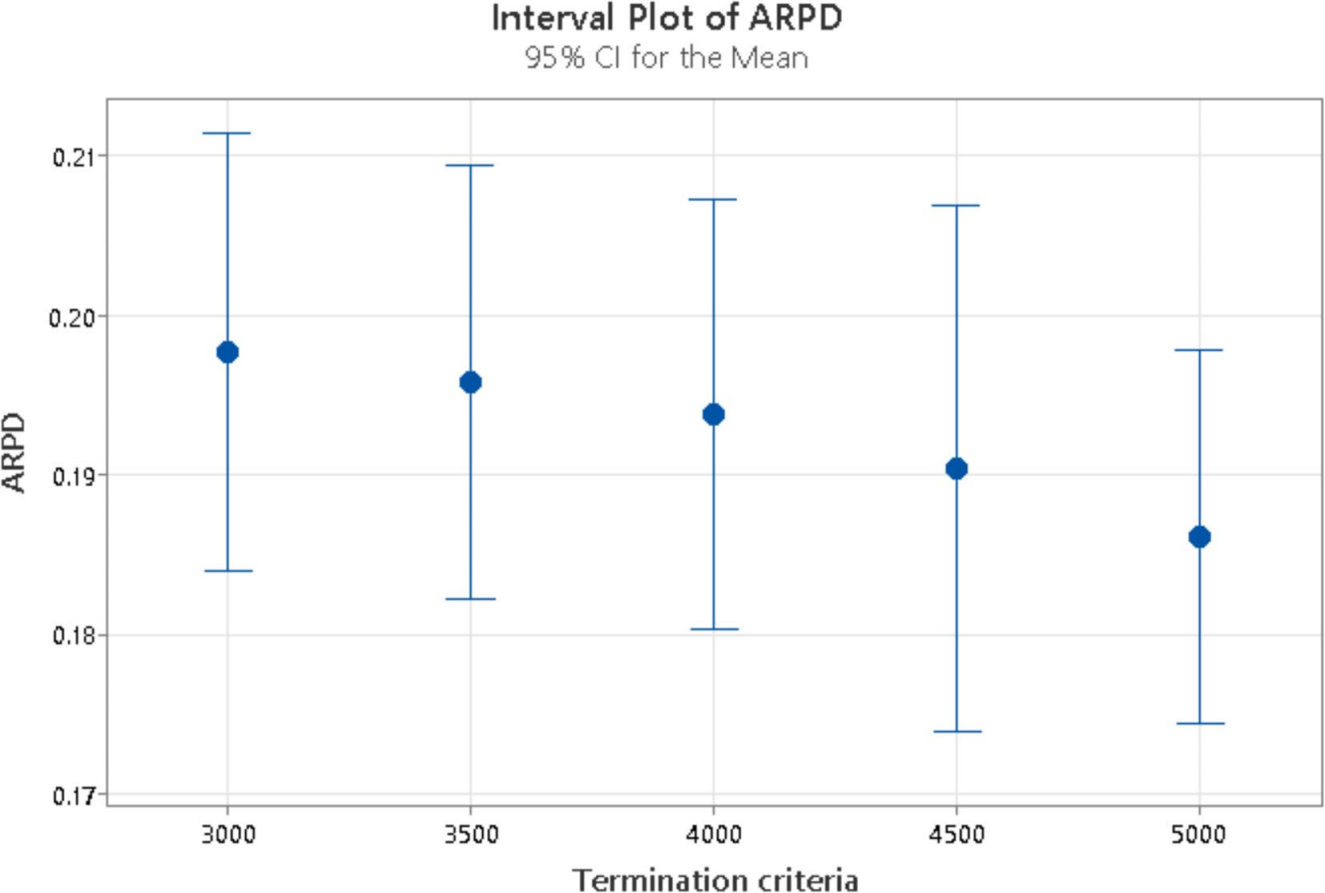
Boxplot for termination criteria.

The results in Table 3 show the P-value for the reproduction operator is 0.000, hence it is statistically significant as its P-value is less than 0.05. The reproduction operator significantly influences the response variable. In contrast, the P-value associated with mutation type is 0.927, indicating that mutation type does not have a meaningful impact on the response variable. Similarly P-value for termination criteria is 0.680, hence it also has no substantial effect on the response variable and it fails to reject the Null hypothesis. Figures 7, 8 and 9 present the means along with a 95% confidence interval for mutation type, reproduction type, and termination criteria. A statistically significant difference between two means is indicated when the ARPD values for those means do not overlap.

Statistically, the value of reproduction operator 5 is better than 4, 8 is better than 5, 9 is better than 8, and 16 is better than 9, as shown in Fig. 8. However, out of all the reproduction operators, value 16 is the best. From Fig. 7 on mutation rates, we can see that a double swap is better than a single swap and a quad swap is better than a double swap. Therefore, we select the quad-swap mutation operator. Regarding the termination criteria, Fig. 9 shows that the ARPD value at 5000 iterations is the lowest when compared to values at 3000, 3500, 4000, 4500, and 5000 iterations. As a result, we set the reproduction operator to 16 and the mutation rate to quad-swap mutation, along with a 5,000 iteration count.

## Computational results

### Test problems

To assess the effectiveness of the HES algorithm, they are tested on benchmark problems of Demirkol, et al.^7^. Demirkol instances are hard instances and are used in experimental results by numerous researchers. The Demirkol benchmark problems can be downloaded using URL^78^. A total of 600 problems are proposed by Demirkol, et al.^7^ for shop scheduling problems. 120 instances are for minimization of makespan (C_max_) while 480 instances are for minimization of maximum Lateness (L_max_). 40 instances are used for the minimization of C_max_ for NPFSSP. For these instances, machine ranges are 15 & 20, while job ranges are 20, 30, 40 & 50. Hence, the total number of combinations is eight, and the number of operations ranges from 300 to 1000. For all these eight combinations, a total of ten instances are generated.

### Results and discussion

The performance of any algorithm is dependent on the exact tuning of its genetic operators. NEH heuristic is utilized to generate the initial solution, which is then refined using the global search ability of the ES algorithm. To guide the ES to less explored areas and to escape local minima, Hybridization is performed by incorporating a frequency table and Local Search Technique. HES is coded in MATLAB 2013 and runs on a Core™ i5 with a 2.4 GHz processor with 8 GB RAM. To validate the results of the HES, it has been run 30 times for each instance of Demirkol benchmark problems. The computational time and makespan of each instance tested on the HES algorithm are shown in Table 2. The solution of the HES is compared with the Shifting bottleneck heuristic of Demirkol, et al.^7^ (DMU). Ying and Lin^15^ proposed a Multi-heuristic Desirability Ant Colony algorithm (MHD-ACS) and improved the upper bound for 32 out of 40 problems of Demirkol. The MHD-ACS algorithm was coded in Visual C + + and run on a Pentium 4 (1.5 GHz) Processor. Each instance was run five times and the termination criteria were 5000 iterations. Rossi and Lanzetta^18^ suggested a Native Non-Permutation Ant Colony Optimization (NNP-ACS) algorithm to solve the NPFSSP of DMU. The NNP-ACS algorithm runs with a Pentium 4 (3 GHz) processor and 2 GB RAM. Each instance was run ten times and the termination criteria were 3000 iterations. Ying^17^ proposed an IG algorithm for NPFSSP and validated his results on Demirkol benchmark problems. The IG algorithm was coded in Visual C# and ran a Pentium 4 (2.4 GHz) processor. Cui, et al.^20^ suggested a Hybrid GA (HIGA) for NPFSSP with unavailability constraints. The HIGA algorithm was coded in C# and ran an Intel Core i3 3.3 GHz and 4 GB RAM.

To fairly compare HES with the other algorithms i.e. DMU, MHD-ACS, NNP-ACS, IG, HIGA all these algorithms were coded in MATLAB 2013 and run on the same processor, i.e. Core™ i5 with a 2.4 GHz processor with 8 GB RAM. Termination criteria for all these algorithms were also set at 5000 iterations. Makespan values and computational time taken by DMU, MHD-ACS, NNP-ACS, IG, and HIGA for Demirkol problems are shown in Table 3. From Table 3 it is evident that the computational time taken by HES for all the 40 Demirkol problems is also lower than the computational time taken by DMU. Thus, in terms of processing time and solution quality, HES has surpassed DMU. HES also outperforms MHD-ACS for all 40 instances in terms of makespan values and computational time. HES found New 40 UB for all Demirkol problems and performed better than NNP-ACS for all 40 problems. However, the computational time taken by NNP-ACS is much higher than the computational time of HES. For the instance, DMU_20_15_3, the computational time of ES is 15.88 s, while for NNP-ACS it is 45.71, which is almost 3 times more than HES. For the instance, DMU_30_15_3, the computational time of HES is 17.31 s while for NNP-ACS it is 178.78 which is almost ten times the computational time of HES. For the instance, DMU_50_20_2, the computational time of HES is 28.58 s while for NNP-ACS it is 719.18, which is almost twenty-five the computational time of HES. Hence, it is conceived that HES performs better than NNP-ACS in terms of computational time and makespan values. HES performs better than IG for all 20 jobs problems although the computational time taken by IG is less. However, looking at the solution quality the computational time difference is very small and can be ignored. For 30 jobs with 15 and 20 machines, HES performs better than IG. For 40 jobs with 15 and 20 machines, IG performs better than HES and the computational time taken by IG is almost twice the computational time of HES. For 50 jobs and 15 machines case, the performance of IG surpasses that of HES. Conversely, in a situation involving 50 jobs and 20 machines, HES demonstrates superior performance compared to IG. Furthermore, the computational time required by IG exceeds twice that of HES across all instances involving 50 jobs.

HES finds a better solution as compared to HIGA for problems with 50 jobs and 15 machines. For 20 jobs and 20 machines cases, HES finds a better solution, and the computational time taken by HES is almost the half of computational time of HIGA. In the case of having 30 jobs and 15 machines, HIGA performs better than HES but at the expense of computational time, as the computational time taken by HIGA is thrice the computational time of HES. In the case of having 30 jobs and 20 machines, HES performs better than HIGA and the computational time of HES is minimum. In the case of 30 jobs and 15 machines, HIGA performs better than HES but at the expense of computational time, as the computational time taken by HIGA is thrice the computational time of HES. Similar is the case for 30 jobs and 20 machine instances where HIGA performs better than HES. HES outperformed HIGA for all 40 job instances but the computational time taken by HIGA is almost five times as compared to the computational time of HES. For 50 jobs and 15 machines cases, HIGA finds better solutions while in the case of having 50 jobs and 20 machines, HES performs better than HIGA. In terms of the overall comparison of HES with techniques, HES outperforms DMU, MHD-ACS, and NNP-ACS algorithms. The performance of HES is at par with the performance of IG and HIGA, however, the computational time of IG and HIGA is much more as compared to the computational time of HES.

The suggested HES is suitable for both small and large-scale problems, demonstrating significantly reduced computational time in comparison to other algorithms documented in the literature for NPFSSP. Therefore, it is crucial to implement the proposed method in practical scenarios to assess its efficacy and leverage the minimal computational time that HES offers in determining optimal processing sequences.

**Table 3:** Comparison of HES results with other benchmark problems.

Instance	DMU			MHD-ACS		NNP-ACS		IG				HES	
	LB	C_best_	T(s)	C_best_	T(s)	C_best_	T(s)	C_best_	T(s)	C_best_	T(s)	C_best_	T(s)
DMU_20_15_3	3354	4437	26.90	4420	17.69	4047	45.71	3915	10.03	3899	19.61	**3825**	15.88
DMU_20_15_6	3168	4144	27.14	4044	17.69	3950	44.90	3788	10.03	3751	19.61	**3808**	16.16
DMU_20_15_4	2997	3779	22.25	3786	17.69	3692	46.53	3558	10.03	3531	19.61	**3507**	14.39
DMU_20_15_10	3420	4302	25.65	4265	17.31	4176	47.76	4048	10.03	4032	19.61	**4013**	14.66
DMU_20_15_5	3494	4373	22.13	4310	17.31	4097	46.94	3910	10.03	3910	19.61	**3908**	13.44
DMU_20_20_1	3776	4821	61.20	4819	22.69	4790	110.61	4558	13.38	4523	26.14	**4517**	13.49
DMU_20_20_3	3758	4779	57.00	4723	23.08	4694	112.65	4432	13.38	4424	26.14	4520	13.27
DMU_20_20_9	3902	4944	67.59	4922	23.08	4720	115.10	4538	13.38	4520	26.14	**4450**	14.34
DMU_20_20_2	3881	4886	77.92	4847	23.08	4731	115.10	4506	13.38	4496	26.14	4530	13.3
DMU_20_20_10	3823	4717	63.10	4715	23.08	4554	114.29	4382	13.38	4373	26.14	4499	14.11
DMU_30_15_3	4020	5226	57.30	5210	35.77	4927	178.78	4602	22.58	4543	44.12	**4511**	17.31
DMU_30_15_4	4080	5304	62.78	5284	36.15	5033	175.51	4687	22.58	4617	44.12	4699	17.42
DMU_30_15_9	4022	5079	41.78	5075	36.54	4912	180.41	4593	22.58	4554	44.12	4641	17.56
DMU_30_15_8	4490	5605	43.12	5593	36.15	5220	177.14	5002	22.58	4836	44.12	**4931**	17.77
DMU_30_15_6	4184	5147	56.67	5149	35.77	5097	186.12	4815	22.58	4744	44.12	4853	17.56
DMU_30_20_3	4806	6183	176.43	5987	46.54	5794	320.00	5437	30.10	5361	58.82	**5357**	19.95
DMU_30_20_1	4772	6037	181.05	5989	47.69	6179	296.33	5703	30.10	5666	58.82	5705	19.78
DMU_30_20_6	5004	6241	151.67	6195	47.69	6039	320.00	5824	30.10	5815	58.82	**5715**	25.57
DMU_30_20_10	4899	6095	123.16	5923	46.54	5888	307.76	5538	30.10	5440	58.82	**5537**	24.08
DMU_30_20_2	4757	5822	149.38	5840	47.31	5842	325.71	5479	30.10	5354	58.82	**5327**	17.69
DMU_40_15_5	5560	6986	80.93	6972	59.23	6521	440.82	5994	40.13	5949	78.43	6105	26.09
DMU_40_15_9	5119	6351	86.02	6310	59.23	6244	426.53	5745	40.13	5678	78.43	5891	24.81
DMU_40_15_2	5290	6506	98.20	6532	59.23	6302	439.59	5923	40.13	5890	78.43	5975	24.19
DMU_40_15_10	5596	6845	92.35	6712	60.00	6413	432.65	5911	40.13	5929	78.43	6061	25.31
DMU_40_15_8	5576	6783	91.54	6771	60.00	6526	424.49	6092	40.13	6086	78.43	6184	25.28
DMU_40_20_3	5693	7154	236.73	7132	80.77	7208	665.31	6554	53.51	6509	104.58	7201	28.21
DMU_40_20_9	5998	7528	248.15	7496	80.00	7388	678.37	6687	53.51	6665	104.58	7223	27.02
DMU_40_20_6	5990	7469	259.20	7476	80.38	7455	667.76	6871	53.51	6834	104.58	7226	28.2
DMU_40_20_7	6170	7608	262.12	7588	79.62	7405	653.47	6786	53.51	6770	104.58	7272	28.33
DMU_40_20_5	6011	7219	232.98	7217	80.77	7326	658.78	6569	53.51	6520	104.58	7221	26.53
DMU_50_15_6	6290	7673	120.44	7631	91.54	7559	706.53	6855	62.71	6899	122.55	7081	28.71
DMU_50_15_5	6355	7679	114.82	7496	92.31	7317	700.41	7028	62.71	6747	122.55	6901	28.88
DMU_50_15_1	6198	7416	109.18	7402	92.31	7205	720.82	6592	62.71	6570	122.55	6792	29.9
DMU_50_15_8	6312	7548	118.22	7558	91.15	7348	701.22	6841	62.71	6889	122.55	7018	28.7
DMU_50_15_2	6531	7750	136.07	7712	90.77	7547	719.18	7073	62.71	6998	122.55	7228	28.58
DMU_50_20_2	6740	8838	490.77	8836	120.00	8436	995.10	7893	83.61	7678	163.40	7996	28.86
DMU_50_20_1	6736	8539	492.31	8521	120.00	8064	996.73	7358	83.61	7366	163.40	**7303**	31.58
DMU_50_20_7	6756	8417	500.38	8425	120.38	8370	1008.16	7752	83.61	7584	163.40	**7500**	30.64
DMU_50_20_8	6897	8590	507.69	8536	120.38	8430	980.41	7529	83.61	7470	163.40	**7412**	34.26
DMU_50_20_4	6830	8493	514.13	8502	120.00	8538	1008.98	7896	83.61	7737	163.40	**7700**	34.01

**Fig. 10:**
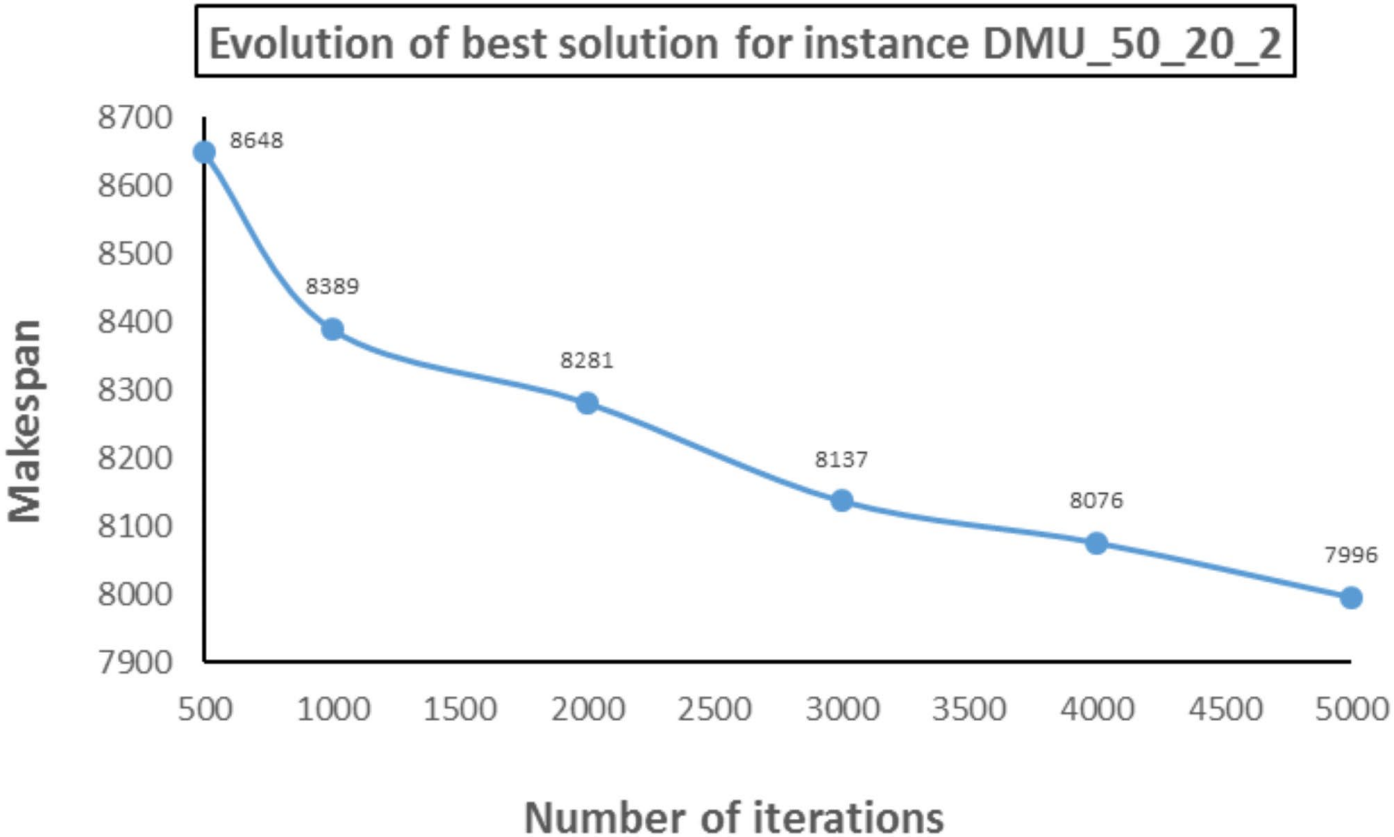
Makespan Comparison of HES with benchmark problems.

**Fig. 11:**
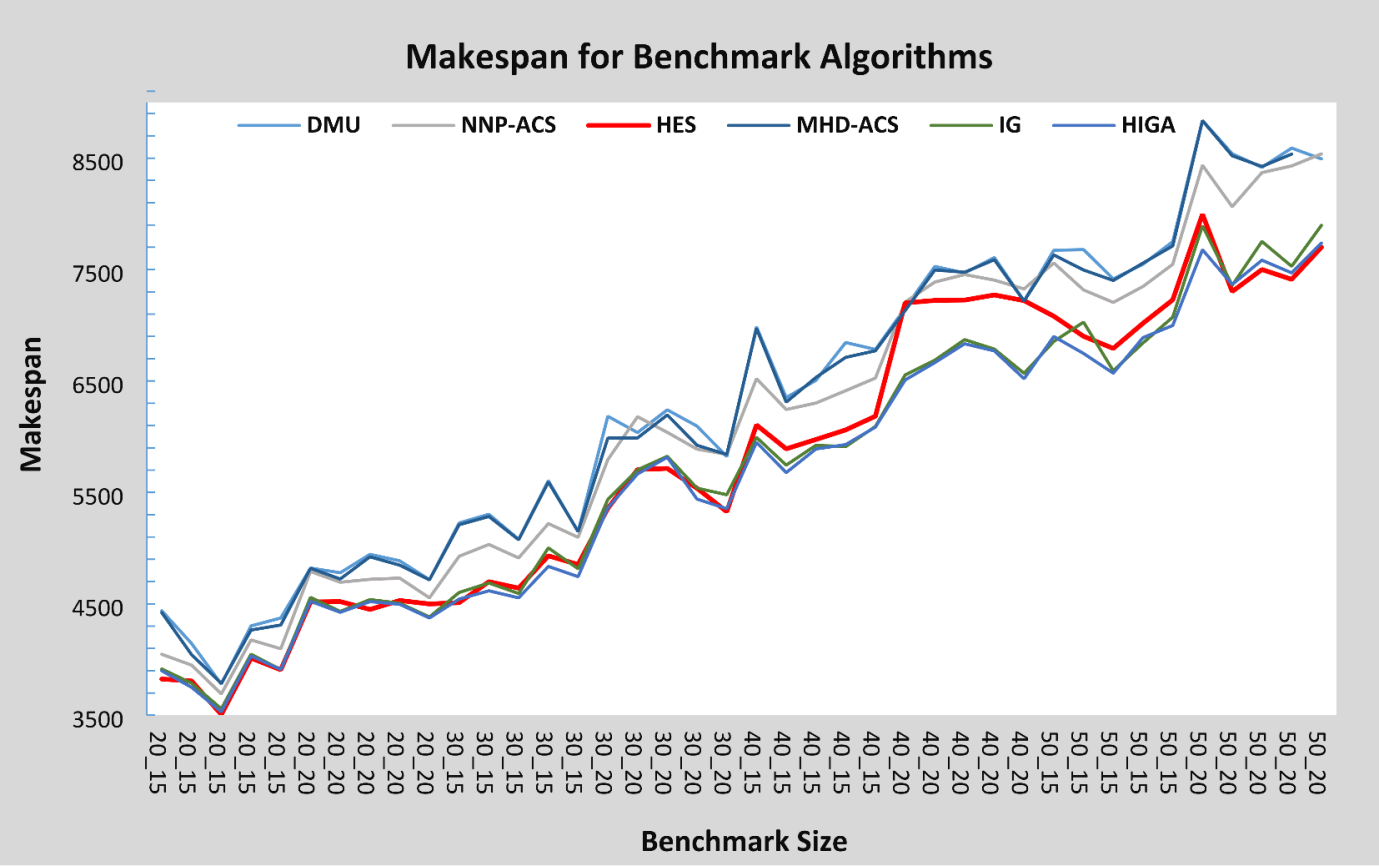
Evolution of the best solution.

Figure 10 provides a comparison of HES with DMU, MHD-ACS, NNP-ACS, IG, and HIGA algorithms in terms of makespan values. From Fig. 10, it ca. be seen that HES performs better than DMU, NNP-ACS, and MHD-ACS. HES finds a better solution as compared to IG and HIGA for 20 and 30 jobs instances, however, for 30 and 40 jobs, IG and HIGA find better makespan values as compared to HES. In the case of 50 jobs and 15 machines, both IG and HIGA find better makespan values. For 50 jobs and 20 machine cases, HES finds better makespan values as compared to IG and HIGA. Another important aspect of the performance of any algorithm is its improvement in objective function value, which increases the number of iterations. In Fig. 11, the graph is shown for the instance DMU_50_20_2, in which the x-axis shows the number of iterations while the y-axis shows the makespan value at the respective number of iterations. At 500 iterations, the makespan is 8648, and at 1000 iterations, it reduces to 8389. Makespan values at 2000, 3000, 4000, and 5000 iterations are 8281, 8137, 8076, and 7996. Hence, there is a continuous improvement in makespan values with the number of iterations. Therefore, it is evident that the HES algorithm avoids local minima and finds better schedules in its neighborhood with an increase in the number of iterations.

The performance of any algorithm is calculated using the % GAP from the lower bound, hence for the Demirkol benchmark instances %GAP can be calculated for all the instances using Eq. (7)

7 $$\:\text{\%}GAP=\:\frac{{C}_{best}-{LB}_{d}}{{LB}_{d}}$$

$$\:where\:$$$$\:{LB}_{d}\:is\:the\:lower\:bound\:found\:by\:Demirkol\:$$$$\:{\:\:\:\:\:\:\:\:\:\:\:\:\:\:\:\:\:\:\:\:\:\:\:\:\:\:\:\:\:\:\:\:\:\:\:\:\:\:\:\:\:\:\:\:\:\:\:\:\:\:\:\:\:\:\:\:\:\:\:\:C}_{\:best}\:is\:the\:best\:makespan\:found\:from\:any\:algorithm$$.

In Table 4, the %GAP for all 40 problems of DMU is calculated using HES, DMU, MHD-ACS, NNP-ACS, IG, and HIGA Algorithms. For instance, DMU_20_15_3, the %GAP for DMU and HES is 32.29% and 14.04%, which is almost half the %GAP of DMU. %GAP for the instance DMU_30_15_3, for DMU and HES, is 30% and 12.21% respectively, so for this instance, the %GAP for HES is less than half the value of DMU. So it shows that HES gives very good results for small instances of problems. Now comparing results of large instances, the %GAP for the instance DMU_50_20_2, for DMU and HES is 18.66% and 10.67% respectively. So even for large instances, HES is giving better results as compared to the results of DMU. Similarly, the %GAP of HES for all 40 instances is less than the %GAP of DMU, which shows the better performance of the HES algorithm for the 40 instances. %GAP calculated by MHD-ACS is better than the %GAP calculated by DMU for all 40 instances, however, it is inferior to the %GAP of HES. %GAP of NNP-ACS is better than both DMU and MHD-ACS, however, it is also inferior to the %GAP values of HES. Therefore, it is proved that the performance of HES is better than DMU, MHD-ACS, and NNP-ACS in terms of %GAP values. %GAP values of HES are at par with the HIGA however IG provides better values, especially for instances where jobs are 40 and 50 and the machines range from 15 to 20.

The overall average %GAP for DMU, MHD-ACS, NNP-ACS, IG, HIGA, and HES is 25%, 21.18%, 24.27%, 13.35% 4.53%, and 14.53% respectively. So in terms of overall performance HES is better than DMU, MHD-ACS, NNP-ACS, and at par with HIGA however, IG performs better than HES in terms of average %GAP values.

**Table 4:** Comparison of %GAP of HES with other benchmark problems.

Instance	DMU	MHD-ACS	NNP-ACS	IG	HIGA	HES
	%GAP	%GAP	%GAP	%GAP	%GAP	%GAP
DMU_20_15_3	32.29%	31.78%	20.66%	16.73%	14.04%	14.04%
DMU_20_15_6	30.81%	27.65%	24.68%	19.57%	20.20%	20.20%
DMU_20_15_4	26.09%	26.33%	23.19%	18.72%	17.02%	17.02%
DMU_20_15_10	25.79%	24.71%	22.11%	18.36%	17.34%	17.34%
DMU_20_15_5	25.16%	23.35%	17.26%	11.91%	11.85%	11.85%
DMU_20_20_1	27.67%	27.62%	26.85%	20.71%	19.62%	19.62%
DMU_20_20_3	27.17%	25.68%	24.91%	17.94%	20.28%	20.28%
DMU_20_20_9	26.70%	26.14%	20.96%	16.30%	14.04%	14.04%
DMU_20_20_2	25.90%	24.89%	21.90%	16.10%	16.72%	16.72%
DMU_20_20_10	23.38%	23.33%	19.12%	14.62%	17.68%	17.68%
DMU_30_15_3	30.00%	29.60%	22.56%	14.48%	12.21%	12.21%
DMU_30_15_4	30.00%	29.51%	23.36%	14.88%	15.17%	15.17%
DMU_30_15_9	26.28%	26.18%	22.13%	14.20%	15.39%	15.39%
DMU_30_15_8	24.83%	24.57%	16.26%	11.40%	9.82%	9.82%
DMU_30_15_6	23.02%	23.06%	21.82%	15.08%	15.99%	15.99%
DMU_30_20_3	28.65%	24.57%	20.56%	13.13%	11.46%	11.46%
DMU_30_20_1	26.51%	25.50%	29.48%	19.51%	19.55%	19.55%
DMU_30_20_6	24.72%	23.80%	20.68%	16.39%	14.21%	14.21%
DMU_30_20_10	24.41%	20.90%	20.19%	13.04%	13.02%	13.02%
DMU_30_20_2	22.39%	22.77%	22.81%	15.18%	11.98%	11.98%
DMU_40_15_5	25.65%	25.40%	17.28%	7.81%	9.80%	9.80%
DMU_40_15_9	24.07%	23.27%	21.98%	12.23%	15.08%	15.08%
DMU_40_15_2	22.99%	23.48%	19.13%	11.97%	12.95%	12.95%
DMU_40_15_10	22.32%	19.94%	14.60%	5.63%	8.31%	8.31%
DMU_40_15_8	21.65%	21.43%	17.04%	9.25%	10.90%	10.90%
DMU_40_20_3	25.66%	25.28%	26.61%	15.12%	26.49%	26.49%
DMU_40_20_9	25.51%	24.97%	23.17%	11.49%	20.42%	20.42%
DMU_40_20_6	24.69%	24.81%	24.46%	14.71%	20.63%	20.63%
DMU_40_20_7	23.31%	22.98%	20.02%	9.98%	17.86%	17.86%
DMU_40_20_5	20.10%	20.06%	21.88%	9.28%	20.13%	20.13%
DMU_50_15_6	21.99%	21.32%	20.17%	8.98%	12.58%	12.58%
DMU_50_15_5	20.83%	17.95%	15.14%	10.59%	8.59%	8.59%
DMU_50_15_1	19.65%	19.43%	16.25%	6.36%	9.58%	9.58%
DMU_50_15_8	19.58%	19.74%	16.41%	8.38%	11.19%	11.19%
DMU_50_15_2	18.66%	18.08%	15.56%	8.30%	10.67%	10.67%
DMU_50_20_2	31.13%	31.10%	25.16%	17.11%	18.64%	18.64%
DMU_50_20_1	26.77%	26.50%	19.71%	9.23%	8.42%	8.42%
DMU_50_20_7	24.59%	24.70%	23.89%	14.74%	11.01%	11.01%
DMU_50_20_8	24.55%	23.76%	22.23%	9.16%	7.47%	7.47%
DMU_50_20_4	24.35%	24.48%	25.01%	15.61%	12.74%	12.74%

**Fig. 12:**
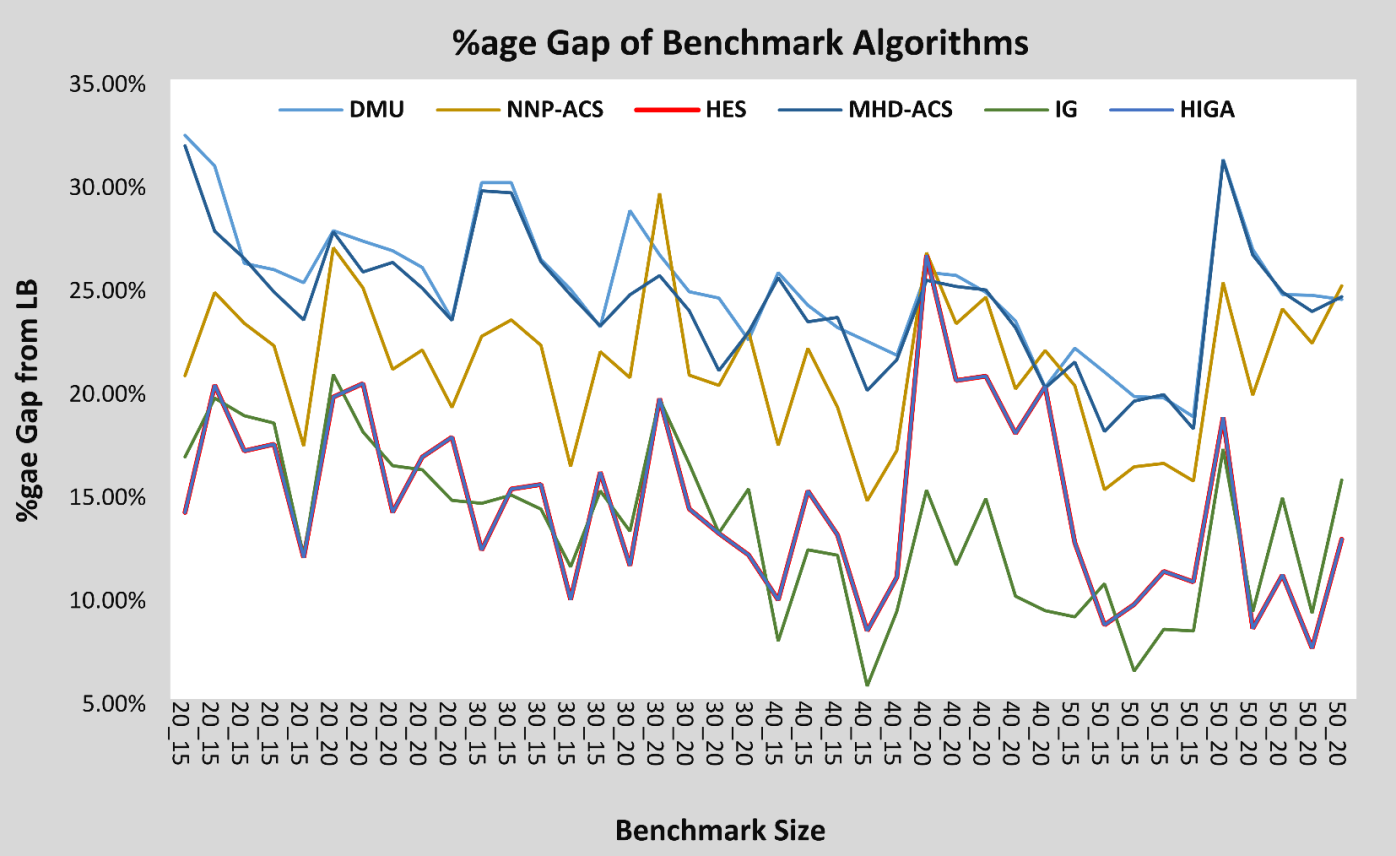
%GAP Comparison of HES with other benchmark problems from LB.

In Fig. 12, the %GAP for DMU, MHD-ACS, NNP-ACS, IG, HIGA, and HES is shown in graphical form. The smaller the %GAP the better the performance of the algorithm, hence from Fig. 12, it is clear that for all the 40 DMU problems, the %GAP of HES is minimum, as compared to the %GAP, found by DMU, MHD-ACS & NNP-ACS. This proves the better performance of HES than DMU, MHD-ACS, and NNP-ACS. The performance of HES is at par with the performance of IG and HIGA.

#### Wilcoxon signed rank test

A non-parametric statistical test called the Wilcoxon signed-rank test is used to assess the effectiveness of two algorithms. Finding out if the paired performance measurements of two algorithms differ statistically significantly across various datasets or experimental settings is its main goal. To assess how well the HES algorithm performs in comparison to the DMU, MHD-ACS, NNP-ACS, IG, and HIGA algorithms, It is essential to assess the statistical differences in the performance of algorithms by employing the Wilcoxon signed rank test. Should the asymptotic significance value fall below the predetermined significance level of 0.05, it indicates a statistically significant difference between A and B. Table 5 displays the results of the Wilcoxon test for the algorithms HES, DMU, MHD-ACS, NNP-ACS, IG, and HIGA. The values of the Alternative Hypothesis and Null Hypothesis for HES versus DMU, MHD-ACS, NNP-ACS, IG, and HIGA, calculated against the Wilcoxon signed test, are shown in Table 6. The Cmax and PI values of HES differ considerably from the DMU method since the P-value for HES vs. DMU is less than 0.05.

**Table 5:** Result of Wilcoxon signed test for HES, DMU, MHD-ACS, NNP-ACS, IG, and HIGA algorithms.

Algorithm pair	Metric	*N*	Median	*P*-value	Wilcoxon statistic	Hypothesis Test	
						Null (H_o_)	Alternative (H_1_)
HES vs. DMU	C_max_ PI	40	−525.75 −0.105	0.00 0.00	3.00 3.00	η = 0 η = 0	η ≠ 0 η ≠ 0
HES vs. MHD-ACS	C_max_ PI	40	−319.00 −0.06	0.00 0.00	0.00 0.00	η = 0 η = 0	η ≠ 0 η ≠ 0
HES vs. NNP-ACS	C_max_ PI	40	−475.75 −0.09	0.00 0.00	3.00 0.00	η = 0 η = 0	η ≠ 0 η ≠ 0
HES vs. IG	C_max_ PI	40	38.00 0.006	0.12 0.168	525.50 513.00	η = 0 η = 0	η ≠ 0 η ≠ 0
HES vs. HIGA	C_max_ PI	40	90.00 0.017	0.00 0.00	688.00 690.00	η = 0 η = 0	η ≠ 0 η ≠ 0

Additionally, the C_max_ and PI values of HES differ considerably from those of the MHD-ACS algorithm since the P-value for HES vs. MHD-ACS is less than 0.05. The C_max_ and PI values of HES differ considerably from those of the MHD-ACS and HIGA algorithms, and similarly, the P-values for HES vs. NNP-ACS and HES vs. HIGA are less than 0.05. Nonetheless, the HES vs. IG P-value is more than 0.05, indicating that the C_max_ and PI values for the two methods do not differ significantly. Therefore, the statistical results of HES are better than DMU, MHD-ACS, NNP-ACS, and HIGA algorithms but inferior to the IG algorithm.

#### Friedman test

A non-parametric statistical test called the Friedman test is used to compare different algorithms on various datasets. Finding out whether there are any appreciable variations in the algorithms’ performance is its main goal. Using a Friedman test, the HES algorithm’s performance is contrasted with the DMU, MHD-ACS, NNP-ACS, IG, and HIGA algorithms’ performances. A statistical technique for assessing how well suggested algorithms perform in comparison to other algorithms is the Friedman test. Table 6 displays the average rating of all algorithms in comparison to five algorithms. ARPD data are used to compare each algorithm. The IG algorithm, with a mean rank of 1.88, outperforms all other algorithms considered. Following closely, the HES-SA algorithm holds the second position with a mean rank of 2.13. Additionally, the performance of the HIGA algorithm is comparable to that of the HES algorithm. Other algorithms i.e. NNP-ACS, MHD-ACS, and DMU have mean ranks of 4.24, 5.06, and 5.58 respectively and their performance is inferior as compared to the HES algorithm.

**Table 6:** Friedman test based on ARPD values.

Algorithm	Mean rank
DMU	5.58
MHD-ACS	5.06
NNP-ACS	4.24
IG	1.88
HIGA	2.13
Proposed method (HES algorithm)	2.13

## Conclusion and recommendations

Since the objective of this research is the minimization of makespan for NPFSSP, hence schedules are generated for NPFSSP using a HES algorithm. Researchers have dedicated significant attention to Hybrid Meta-heuristics over the years, aiming to integrate the most effective characteristics of various methodologies. In this context, the HES algorithm merges the exploitation capabilities of the NEH heuristic with the exploratory aspects of the IES algorithm. Additionally, to avoid being trapped in local minima, a Local Search Method is integrated into the IES algorithm. The initial solution was generated using the NEH heuristic, which acts as a seed for IES. The mutation rate for the Quad swap is utilized, and adjustments to this rate are made to identify the optimal outcomes while minimizing computational time. To guide the search to promising areas, a frequency table is used, which stores the history of mutated chromosomes and each chromosome can be mutated a maximum of 50 times. The frequency table limits the cyclic repetition of the same mutation chromosomes and then guides the search to less explored areas of the solution space. The algorithm underwent testing on 40 instances of the Demirkol Benchmark problems related to the NPFSSP. The findings indicate that the HES algorithm outperformed other solution methods documented in the literature, demonstrating superior performance in terms of computational time, solution quality, and robustness.

Further recommendations are as follows: First, the algorithm should be applied to real-life problems to minimize their total completion time. Second, the objective of this research was makespan, however, it can be extended to other performance criteria i.e. tardiness, total flow time, maximum lateness, total tardiness, etc. Third, HES can be applied to other manufacturing environments. Fourth, the proposed research did not account for sequence-dependent setup times; however, it is recommended that such factors be incorporated in future studies. Fifth, develop an algorithm to solve NPFSSP with more than three objectives. Sixth, in this paper, the processing times are deterministic; the proposed technique should be extended to problems with stochastic processing times. Finally, schedules are generated for Demirkol problems, however, schedules must be generated for VRF (Vallada, et al.^30^) instances, to validate the performance of HES on harder benchmark problems.

## Data Availability

Data is available upon request from the corresponding author.
